# Unveiling the Demographics and Clinical Characteristics of Diabetic Neuropathy: Findings From the National Health Insurance Service, 2012–2017

**DOI:** 10.1111/1753-0407.70196

**Published:** 2026-04-29

**Authors:** Su Jin Jeong, Sun Keun Kim, Chong Hwa Kim

**Affiliations:** ^1^ Division of Endocrinology and Metabolism, Department of Internal Medicine Sejong General Hospital Bucheon Kyunggido South Korea; ^2^ Department of Internal Medicine Heartbeat Analysis Clinic Sungnam Kyunggido South Korea

**Keywords:** diabetes mellitus, diabetic neuropathy, national health insurance, prevalence

## Abstract

**Background:**

Diabetic neuropathy (DN) is the most common chronic complication of Type 2 diabetes mellitus (T2DM), with reported prevalence ranging from 23% to 54.5%. This study evaluated the prevalence, clinical characteristics, and treatments of DN using Korean National Health Insurance Service (NHIS) data from 2012 to 2017.

**Methods:**

We analyzed NHIS sample data for 8.7 million individuals stratified by age, sex, eligibility, and income. DN was defined using ICD‐10 codes (E10.4–E14.4, G59.0, G63.2, G99.0) and concurrent prescriptions for diabetes and DN. Annual DN prevalence among diabetes patients was calculated, and treatment patterns and patient characteristics were compared between those with and without DN.

**Results:**

DN prevalence declined from 23.4% in 2012 to 21.5% in 2017. About half of DN patients received pharmacologic treatment—mainly monotherapy (up to 82%), followed by dual (15%) and triple therapy (3%). The most prescribed drugs were α‐lipoic acid (52.1%–55.0%), anticonvulsants (30.4%–34.5%), tricyclic antidepressants, SNRIs, and γ‐linolenic acid. DN patients were generally older, more often female, and had more comorbidities such as hypertension, dyslipidemia, cardiovascular disease, diabetic foot, and amputations. They were also more likely to use insulin or multiple oral agents.

**Conclusion:**

About one‐quarter of patients with T2DM had DN, and half received treatment, mostly *α*‐lipoic acid monotherapy. DN patients tended to be older and had multiple comorbidities, resulting in higher hospitalization rates.

## Highlights

- Large‐scale analysis of diabetic neuropathy using Korean National Health Insurance data (2012–2017).
- Identified demographic patterns and clinical characteristics across diverse patients group.
- Findings highlight age, sex, and comorbidity as significant factors influencing disease burden.
- Results provide evidence to inform public health strategies and clinical management in Korea.

## Graphical abstract

Diabetic neuropathy prevalence declined from 23.4% to 21.5% between 2012 and 2017. About half received treatment, mainly α‐lipoic acid monotherapy; patients were older with comorbidities, leading to more hospitalizations.

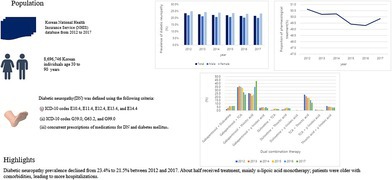

## 1 Introduction

Globally, the prevalence of diabetes is 14.3%, and the death rate due to diabetes is estimated to be 8.4% [1, 2]. Diabetes is also increasing in the population in Korea, and according to statistics on the cause of death by Statistics Korea in 2014, 20.7 deaths per 100,000 people were due to diabetes, accounting for about 3.9% of all deaths [3].

Diabetic neuropathy (DN) is the most common chronic complication among microvascular complications of diabetes and has significant clinical significance such as decreased quality of life and increased morbidity and mortality. However, there are many limitations in diagnosis and treatment in an actual clinical environment because the changes in the nervous tissue and the expression of clinical symptoms in diabetic patients are very diverse. When the pathological phenomenon progresses to a certain level and reaches a clinically verifiable stage, there is a limit to fundamentally reversing the pathological change, so it has no choice but to treat with symptomatic or conservative therapy. Therefore, the clinical characteristics and early screening of DN are important [4].

The prevalence of DN varies from 23% to 50% according to many previous epidemiologic studies [5, 6, 7, 8]. Even studies in the Korean population presented a variety of prevalence from 14.1% to 54.5%. The prevalence of DN in Korea was reported as 33.5% in a nationwide hospital‐based multicenter study by the DN Study Group of the Korean Diabetes Association [6]. However, the hospital‐based epidemiologic study has a limitation to represent the real status of the whole Korean diabetic population. Recently, the annual prevalence of DN was from 24.9% in 2006 to 20.8% in 2015 by the DN Study Group of the Korean Diabetes Association using National Health Insurance Service‐National Sample Cohort (NHIS‐NSC) database [8].

However, epidemiological studies on the basic clinical characteristics and treatment patterns of DN patients are still lacking. Therefore, in this study, the prevalence and treatment patterns of DN in diabetic patients were identified using the National Health Insurance Corporation sample cohort data, and clinical characteristics according to the presence or absence of DN were investigated.

## 2 Methods

The Korean National Health Insurance Service (KNHIS) is a compulsory single‐payer national health care insurance system that covers 98% of the population residing within the territory of South Korea and manages all health service utilization databases [9, 10].

For this study, we selected individuals aged ≥ 30 years with diabetes from 2012 to 2017. Diabetes was defined as a diagnosis of diabetes (as defined by the 10th edition of the International Classification of Diseases [ICD‐10] codes E10−E14) on more than two occasions, receipt of a prescription for oral glucose‐lowering drugs (Anatomical Therapeutic Chemical [ATC] code A10B) for > 30 days, or receipt of an insulin (ATC code A10A) prescription on an outpatient basis.

We defined the presence of DN as follows: (1) a diagnosis of DN (ICD‐10 codes E10.4, E11.4, E12.4, E13.4, E14.4, G59.0, G63.2, and G99.0), or (2) receipt of a prescription for glucose‐lowering drugs (ATC code A10A) plus drugs for DN (ATC codes A16AX01, D11AX02, N06AA10, N06AA09, N03AX12, N03AX16, and N06AX21). Peripheral arterial occlusive disease (PAOD) and lower‐extremity amputation (LEA) were defined as follows: (1) PAOD, procedure codes HA633, HA651, HA652, M6597, M6605, M6612, M6613, M6620, M6632, M6633, N0571‐N0575, O0161‐O171, O1643‐O1646 (2) LEA, procedure codes N0571‐N0575. Crude prevalence rates were calculated as percentages among individuals with diabetes. Age‐adjusted prevalence rates were standardized against the year 2005 weighted distribution of study people with diabetes across sexes and five‐year age groups from 30–34 to 85 years and over.

Among the subjects of the 2012–2017 sample cohort of the National Health Insurance Service (NHIS), the prevalence rate of subjects defined as DN patients was coded diagnosis, treatment drug use patterns were identified, and clinical characteristics according to the presence or absence of DN in diabetic patients were compared.

Database analyses were done for prevalence of DN and reflected independent variables age, sex, hypertension, dyslipidemia, heart diseases, cerebrovascular diseases, and other CVDs. As demographic characteristics, gender, age, and characteristics of major visited medical institutions were calculated. In the health insurance claim data, information such as diagnosis name, drug prescription, hospitalization and discharge, and death record, blood sugar record, insulin and hypoglycemic agent prescription, DN drug, antihypertensive drug, and antihyperlipidemic drug were used; non‐reimbursed drug information was not incorporated in the analysis.

According to this process, a retrospective demographic analysis was performed on 705,721 people, excluding 227,485 people under the age of 30, among 933,206 people diagnosed with diabetes out of 8,696,746 people in the National Health Insurance Corporation sample cohort. Using the prospective cohort data (National Health Insurance Corporation sample cohort), the prevalence of DN patients and the pattern of use of treatment agents were investigated, and the clinical characteristics of diabetic patients according to the presence or absence of DN were compared.

The study protocol was reviewed and approved by the Institutional Review Board of Sejong General Hospital (IRB No. BSH2020‐10‐042).

Pharmacologic treatment for DN was defined as the receipt of a prescription of any medication for DN at least once per year. Continuous treatment was defined as receipt of prescribed medication for DN for at least 290 days (80%) per year. In cases where the medication prescribed was changed several times, the prescription with the longest duration each year was taken to be representative. Medications for DN were classified as anti‐convulsive agents (*α*2*δ* ligands, gabapentinoids), serotonin–norepinephrine reuptake inhibitors (SNRIs, duloxetine), tricyclic antidepressants (TCAs), *α*‐lipoic acid (thioctic acid), and gamma‐linolenic acid (GLA).

### 2.1 Statistical Method

The age‐standardized rate of DN patients by year was calculated through demographic data analysis, and treatment patterns of DN patients by year were summarized. In addition, demographic characteristics and baseline values such as gender, age, comorbidities, obesity, cholesterol, and lifestyle, such as phase indicators according to the presence or absence of DN, were summarized.

All data in this study are anonymized National Health Insurance Corporation sample cohort data, so they cannot be individually identified. They are accessed using the designated National Health Insurance Corporation remote system, and all usage records such as data collection, purification, and analysis are kept. Research ethics and personal information protection were reviewed by both the research institute and the data provider, and the scope and use of data required for research were determined and monitored.

The analysis was conducted using SAS version 9.4 (SAS Institute Inc., Cary, NC, USA).

## 3 Results

### 3.1 Prevalence of DN

**FIGURE 1:**
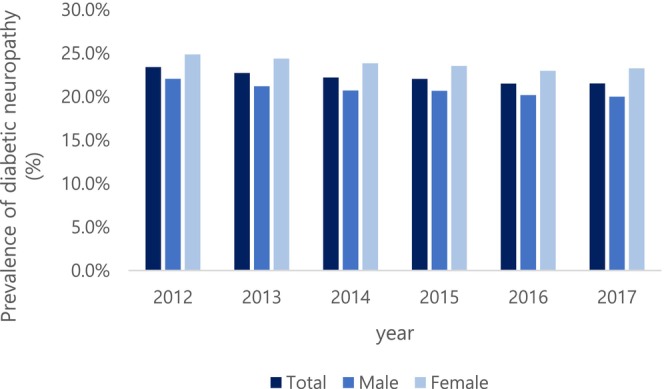
Prevalence of diabetic neuropathy in total study population by gender.

The prevalence of DN in DM population was gradually decreased from 23.4% in 2012 to 21.5% in 2017. For gender, the same trend was observed, decreasing from 24.8% to 23.3% in female and from 22.0% to 20.0% in male (Figure 1). The prevalence of DN in DM population by age was analyzed by stratifying the patient groups meeting the above criteria by age and showed a decrease from 19.0% to 15.4% in the 40 s, 22.0% to 19% in the 50 s, and 25.3% to 22.3% in the 60 s. Visiting institutions by year of diabetic patients were analyzed. Nursing institution variable extraction criteria were set based on the most frequently visited institution for each patient. For general hospitals, it decreased from 27.4% to 21.2%, and for hospitals, from 26.5% to 24.7%, and for clinics, from 27.3% to 24.5%.

### 3.2 Status of Pharmacological Treatment in DN

**FIGURE 2:**
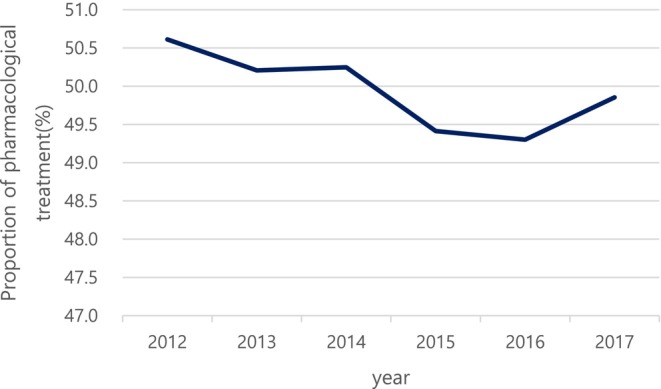
Proportion of pharmacological treatment in diabetic neuropathy.

**FIGURE 3:**
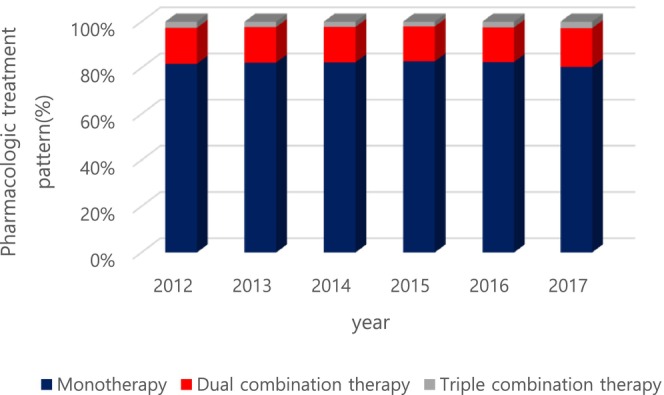
Trends of pharmacologic treatment for diabetic neuropathy.

**FIGURE 4:**
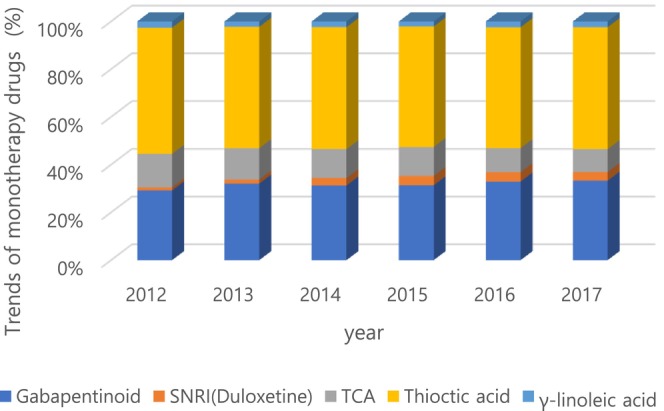
Trends of monotherapy drugs for diabetic neuropathy. Gabapentinoid (gabapentin, pregabalin), thioctic acid (*α*‐lipoic acid), TCT (tricyclic antidepressants), SNRI (serotonin‐norepinephrine reuptake inhibitors), and *γ*‐linolenic acid.

**FIGURE 5:**
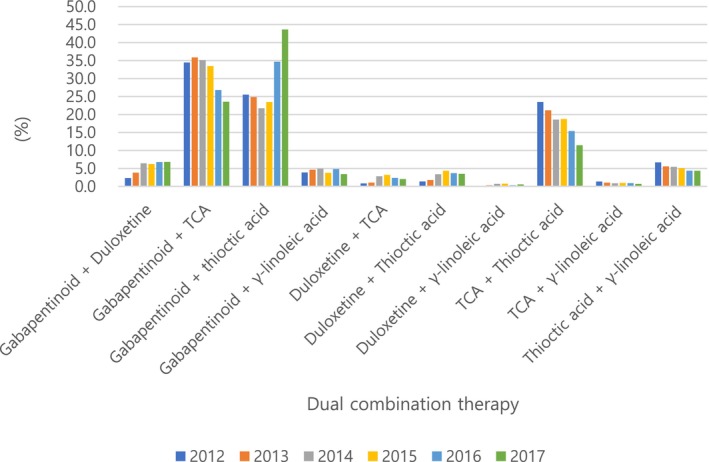
The Trend of Dual combination therapy in diabetic neuropathy. Gabapentinoid (gabapentin, pregabalin), thioctic acid (*α*‐lipoic acid), TCA (tricyclic antidepressants), Duloxetine (SNRI, serotonin‐norepinephrine reuptake inhibitors), and *γ*‐linolenic acid.

The percentage of pharmacological treatment was 50.6% among patients with DN in 2012. Most of the patients with DN (49.4%) did not get prescribed. Through the follow‐up period, the pharmacological prescription rate was between 49.3% and 50.6% where it decreased from 50.6% in 2012 to 49.3% in 2016 and thereafter, the rate increased to 49.9% in 20l7 (Figure 2). Monotherapy, dual therapy, and triple therapy accounted for 80.4%, 15.3%, and 2.7% of total prescriptions in 2012, respectively (Figure 3). Among several classes of drugs, thioctic acid was prescribed the most commonly, and anti‐convulsive agents and tricyclic antidepressants were the second and the third most common drugs, consecutively (Figure 4). However, the prescription of anti‐convulsive agents and SNRI were consistently increasing through the follow‐up period while thioctic acid, tricyclic antidepressants and gamma‐linolenic acid were gradually decreasing. Through the follow‐up period, dual combination therapy accounted for 15.3%–16.5% of total prescriptions. The combination of thioctic acid and tricyclic antidepressants was the most common through 2012–2015 but consistently declining during the follow‐up period. The combination of anti‐convulsive agents and thioctic acid was the most common through 2016–2017 and then increasing (Figure 5).

### 3.3 The Characteristics of Patients With Diabetic Neuropathy

Demographic analysis was conducted on a cohort of 705,721 patients, revealing notable differences between DN and non‐DN diabetic patient groups.

In the gender group, non‐DN diabetic patients predominantly comprised males (53.3%), whereas females were more common in the DN group (51.0%), highlighting a higher prevalence of DN among females compared to males (49.0%).

In the non‐DN diabetic group, age distribution was as follows: 30–39 (3.7%), 40–49 (12.4%), 50–59 (26.7%), 60–69 (27.2%), 70–79 (24.0%), and ≥ 80 (6.1%). In contrast, DN patients were predominantly older, with proportions of 1.9% (30–39), 8.7% (40–49), 23.7% (50–59), 29.1% (60–69), 29.9% (70–79), and 6.8% (≥ 80), indicating increased DN prevalence with advancing age.

Comorbidities were more frequent among DN patients compared to non‐DN diabetic patients: Hypertension: 74.2% versus 68.5%, dyslipidemia: 78.9% versus 74.6%, heart diseases: 77.8% versus 71.8%, cerebrovascular diseases: 22.1% versus 13.7%, other cardiovascular diseases: 41.6% versus 25.1%.

The proportion undergoing diabetic foot surgery was higher in DN patients (1.2%) compared to non‐DN diabetic patients (0.3%) (Table 1).

**TABLE 1:** Demographic and clinical characteristics of adults with diabetes aged ≥ 30 years (NHIS sample cohort, 2012–2017).

Variables	Diabetic neuropathy‐YES (*N* = 156,471) N	Diabetic neuropathy‐YES (*N* = 156,471) %	Diabetic neuropathy‐No (*N* = 549,250) N	Diabetic neuropathy‐No (*N* = 549,250) %	*p*
Sex					**< 0.001**
Male	76,622	49.0	292,891	53.3	
Female	79,849	51.0	256,359	46.7	
Age group					**< 0.001**
30–39	2,980	1.9	20,068	3.7	
40–49	13,571	8.7	68,187	12.4	
50–59	37,007	23.7	146,392	26.7	
60–69	45,584	29.1	149,380	27.2	
70–79	46,733	29.9	131,859	24.0	
80+	10,596	6.8	33,364	6.1	
Type of hospital					**< 0.001**
General hospital	1,017	0.6	2,971	0.5	
Hospital	3,343	2.1	9,805	1.8	
Doctors office	59,472	38.0	170,222	31.0	
etc	74,868	47.8	219,474	40.0	
Diseases					
Hypertension	116,164	74.2	376,098	68.5	**< 0.001**
Dyslipidemia	123,491	78.9	409,776	74.6	**< 0.001**
Heart diseases	121,665	77.8	394,513	71.8	**< 0.001**
Cerebrovascular diseases	34,631	22.1	75,325	13.7	**< 0.001**
Other cardiovascular diseases	65,133	41.6	137,994	25.1	**< 0.001**
Surgery for diabetic foot					**< 0.001**
Yes	1,938	1.2	1,904	0.3	
No	154,533	98.8	547,346	99.7	
Treatment of DM					
Glucose lowering drug	133,492	85.3	389,657	70.9	**< 0.001**
Insulin	34,831	22.3	50,785	9.2	**< 0.001**
Treatment of Diabetic neuropathy					**< 0.001**
1	63,042	40.3	—	—	
2	12,320	7.9	—	—	
3+	2,744	1.8	—	—	

*Note:* Bold values indicate statistical significance (*p* < 0.05) for the presence or absence of diabetic neuropathy.

Abbreviations: DM, Diabetes Mellitus; NHIS: National Health Insurance Service.

The adjusted OR value applied with univariate and multivariate logistic regression analysis was calculated and arranged. Multivariate logistic regression analysis identified significant predictors of DN: Female gender: OR = 1.118 (95% CI: 1.105–1.131), increasing age groups: OR progressively rose from 1.361 (95% CI: 1.303–1.422) for ages 40–49 to 1.876 (95% CI: 1.800–1.956) for ages 70–79, number of hospitalizations: OR = 1.039 (95% CI: 1.036–1.042), history of diabetic foot surgery: OR = 1.726 (95% CI: 1.614–1.846), insulin use: OR = 2.472 (95% CI: 2.433–2.512), dyslipidemia: OR = 1.183 (95% CI: 1.167–1.200), cerebrovascular disease: OR = 1.390 (95% CI: 1.369–1.411), other cardiovascular diseases: OR = 1.888 (95% CI: 1.865–1.911).

However, baseline hypertension (OR = 1.025, CI: 0.993–1.058) and heart disease (OR = 1.011, CI: 0.978–1.046) did not show statistically significant associations with DN (Table 2).

**TABLE 2:** Multi‐logistic regression analysis of odds ratio of factors in diabetic neuropathy.

Variables	Category	Crude OR	LCI (crude)	HCI (crude)	Adjusted OR	LCI (Adj.)	HCI (Adj.)
Sex	Male	1.000			1.000		
	Female	1.191	1.177	1.204	1.118	1.105	1.131
Age group	30–39	1.000			1.000		
	40–49	1.340	1.284	1.399	1.361	1.303	1.422
	50–59	1.702	1.635	1.772	1.622	1.557	1.690
	60–69	2.055	1.975	2.139	1.788	1.716	1.863
	70–79	2.387	2.293	2.484	1.876	1.800	1.956
	≥ 80	2.139	2.046	2.235	1.595	1.523	1.670
Number of admission	No	1.000			1.000		
	Yes	1.090	1.087	1.093	1.039	1.036	1.042
Surgery for diabetic foot	No	1.000			1.000		
	Yes	3.605	3.383	3.842	1.726	1.614	1.846
Insulin	No	1.000			1.000		
	Yes	2.811	2.769	2.853	2.472	2.433	2.512
Hypertension	No	1.000			1.000		
	Yes	1.327	1.310	1.344	1.025	0.993	1.058
Dyslipidemia	No	1.000			1.000		
	Yes	1.274	1.257	1.292	1.183	1.167	1.200
Heart diseases	No	1.000			1.000		
	Yes	1.371	1.353	1.389	1.011	0.978	1.046
Cerebrovascular diseases	No	1.000			1.000		
	Yes	1.788	1.763	1.814	1.390	1.369	1.411
Other cardiovascular diseases	No	1.000			1.000		
	Yes	2.125	2.100	2.150	1.888	1.865	1.911

## 4 Discussion

In this study, the prevalence for the period included 2012–2017 ranged from 21.5% to 23.4%. This result was consistent with the trend of the previous study, which showed a decrease range of 26.6% to 20.8%. Although it is not a diagnosis based on actual clinical DN diagnostic criteria and neurological examination, it is meaningful in that it shows consistent results when defined based on a diagnosis code or drug prescription prescribed in the actual clinic.

There are several theories possible for the decreasing trend of the prevalence of DN. First, since this study is a population study, it could not be matched with personal medical data. However, as the number of people who regularly receive national health checkups is increasing and the diagnosis of diabetes is made at an early time, it can be considered because of the decrease in the duration of disease in the pathophysiological approach of DPN that occurs due to the long‐term prevalence of diabetes.

HbA1c, a glycated hemoglobin, is increasingly being followed, and it can be considered a positive effect of monitoring a specific indicator as a representative example of patient monitoring that contributes to long‐term prognosis.

In addition, the emergence and increase in application of new classes of diabetes drugs, such as GLP1 agonists and SGLT2 inhibitors, have an effect of improving the long‐term prognosis of various diseases, and it can be considered that the drugs have an effect of improving the prognosis as a drug corresponding to the primary indication.

In this study, we noticed that diabetic foot surgery, use of insulin, dyslipidemia, and cardiovascular disease were quantitatively related to the morbidity of DN. In particular, the use of insulin showed a 2.472‐fold increase in the adjusted odds ratio. Recent studies have shown that the insulin signaling pathway actively contributes to sensory neuronal regulation. In the past, muscle and adipose tissue expressed the GLUT4 glucose transporter and absorbed glucose sensitively to insulin, whereas nerve tissue was known to absorb glucose along a concentration gradient through GLUT1 & 3. In a study by Leloup et al., 1996 [11], expression of GLUT4 in specific regions such as the olfactory bulb, hippocampus, and hypothalamus has recently been demonstrated. And this explains the mechanism of damage to the central nervous system by controlling blood sugar and insulin.

In the peripheral nervous system, studies have focused on nervous cell regeneration regulated by insulin signaling in insulin‐dependent diabetes mellitus. Chen et al., 2013 [12] study observed DN symptoms in an insulin‐decreased animal model without hyperglycemia as a recovery pattern following insulin injection, explaining the role of insulin in peripheral nerve recovery. In other words, in insulin‐dependent diabetes mellitus, the correct insulin signal is lost, and the recovery of the insulin signal does not occur at a time when nerve recovery is required. As a result of the statistics, rather than correlating that insulin treatment is related to the prevalence of DN, it should be viewed as a mechanism of insulin‐dependent diabetes mellitus, in which the recovery mechanism of the peripheral nerves controlled by an appropriate insulin signal is lost.

In medical treatment, the ratio of monotherapy was found to be about 80%, and it shows a decreasing trend compared to previous studies, and the rate of treatment with dual and triple drugs increases from 15.2% + 2.3% to 16.5% + 2.8%.

The prescription rate for thioctic acid was 60%, and it became 53%, but it is still most common choice and anticonvulsive agents showed a continuous increase 30.4% to 34.5%, and SNRI also increased continuously. On the other hand, in the case of TCA, it decreased from 14.6% to 9.9%.

The increased use of pregabalin is associated with efficacy demonstrated. As studies (3 Class I and 1 Class II) evaluating the efficacy of pregabalin were reviewed. All studies found that pregabalin relieved pain by 11%–13% compared to placebo on the 11‐point Likert scale in the Class I studies. A large dose‐dependent effect was observed. In the QOL, social functioning, mental health, bodily pain, and vitality improved, and sleep interference decreased [13].

Since duloxetine was also introduced as a guideline drug, the usage increased. In addition to single use, the combined use of duloxetine and other drugs showed a threefold increase. According to three studies (1 Class I and 2 Class II), evaluated the efficacy of duloxetine in DN. The Class I study showed that duloxetine had a small effect compared to placebo, reducing pain by 8% on the 11‐point Likert scale. In 2 Class II studies, duloxetine reduced VAS score 13% more than placebo [13].

Former Nortriptyline and fluphenazine in the symptomatic treatment of DN study of Gomez et al. demonstrated the effectiveness of the two‐drug combination treatment [14]. Afterwards, the number of two‐drug combination treatments increased. However, to date, there have been no studies with head‐to‐head comparison results of combined treatment. If we look at the direction shown by the increase in anti‐convulsive agents(*α*2*δ* ligands) + thioctic acid combination in this study, it can be shown that the prescription of anti‐convulsive agents(*α*2*δ* ligands) + thioctic acid combination is gradually increasing according to clinical experience and significant pain control. It can be a basis for representing the possibility of this. In the future, comparative studies of combination therapy should be conducted for several drugs.

The usage status of classified medical institution was also analyzed and a significant amount of movement was observed from general hospitals to clinics, and movement from general hospitals to clinics and other institutions and from clinics to other institutions was noticed.

Our study is a data analysis study based on populationized data using National Sample Cohort data from the National Health Insurance Corporation. It has the strength of research that can reflect the population group of Korea as it is. Korea's National Health Insurance System is a compulsory social insurance system that covers all citizens and foreigners who have stayed in Korea for more than 6 months and overseas citizens. 98% of the population consists of a single insurer called the National Health Insurance Corporation. As a result, the medical use benefits of all 50 million people can be accumulated in the form of medical practices, materials, drugs, etc. In addition, it is a health check‐up service system for all citizens. There are many countries that operate cancer screening systems, but there are very few places like Korea that measure blood pressure, blood sugar, and even triglycerides and HDL of more than 10 million people every year and accumulate the results [15].

Because health insurance data is easily linked through the resident registration number given to individuals, information such as birth, death, address, workplace, disability, and income of an individual necessary for health insurance operation is linked to the administrative computer network to provide administrative assistance in personal health management [16]. It has the advantage of being easy to do.

On the other hand, since the National Health Insurance Corporation data is based on claim data, non‐reimbursement information is omitted, and only information on whether tests and treatments have been performed can be known, but results are not provided. The most serious drawback is that medical institutions tend to intentionally up‐code the patient's disease severity to prevent cuts in the claim review process [17]. Because of this tendency, simply determining the prevalence of a specific disease based on the health insurance claim symbol leads to overestimating the number of patients. The causal analysis using individual‐level clinical indicators was limited because the NHIS database is population‐based rather than personalized in nature.

Therefore, classification of diseases based on drug use has important meaning. By classifying the disease according to the actual drug use matched with the disease group, it is possible to reduce the confounding variables due to the “up‐coding” mentioned above. Also, depending on the number of drugs to be treated, changes in the severity of the disease can be verifiable. According to the tendency of the single, dual, and triple treatment, the severity of each individual disease can be identified [15, 17].

In conclusion, the prevalence of DN decreased slightly over the 6‐year study period. Thioctic acid monotherapy was the most commonly prescribed treatment. Patients with DN tended to be older and exhibited multiple comorbidities and complications, resulting in higher hospitalization rates. Given the global trend towards aging societies, greater emphasis on early diagnosis and effective management of DN is crucial to mitigate its substantial socioeconomic impact.

## Author Contributions

**Chong Hwa Kim:** conceptualization. **Sun Keun Kim:** data curation. **Su Jin Jeong and Sun Keun Kim:** formal analysis. **Su Jin Jeong and Sun Keun Kim:** writing – original draft. **Su Jin Jeong and Chong Hwa Kim** writing – review and editing.

## Funding

The authors have nothing to report.

## Conflicts of Interest

The authors declare no conflicts of interest.
